# H-rGO-Pd NPs Nanozyme Enhanced Silver Deposition Strategy for Electrochemical Detection of Glypican-3

**DOI:** 10.3390/molecules28052271

**Published:** 2023-02-28

**Authors:** Guiyin Li, Bo Wang, Ling Li, Xinhao Li, Ruijie Yan, Jintao Liang, Xinchun Zhou, Liuxun Li, Zhide Zhou

**Affiliations:** 1 Guangxi Key Laboratory of Information Materials, School of Life and Environmental Sciences, Guilin University of Electronic Technology, Guilin 541004, China; 2 College of Chemistry, Guangdong University of Petrochemical Technology, Guandu Road, Maoming 525000, China; 3 Guangdi Maoming Chemical Co., Ltd., Maoming High-Tech Industrial Development Zone, Maoming 525000, China; 4 Solid Tumour Target Discovery Laboratory, Translational and Clinical Research Institute, Newcastle University Centre for Cancer, Faculty of Medical Sciences, Newcastle University, Newcastle upon Tyne NE2 4HH, UK

**Keywords:** electrochemical nanobiosensor, Glypican-3, H-rGO-Pd NPs nanozyme, peroxidase-like catalytic silver deposition, hepatocellular carcinoma

## Abstract

Glypican-3 (GPC3), as an emerging biomarker, has been shown to be beneficial for the early diagnosis and treatment of hepatocellular carcinoma (HCC). In this study, an ultrasensitive electrochemical biosensor for GPC3 detection has been constructed based on the hemin-reduced graphene oxide-palladium nanoparticles (H-rGO-Pd NPs) nanozyme-enhanced silver deposition signal amplification strategy. When GPC3 specifically interacted with GPC3 antibody (GPC3_Ab_) and GPC3 aptamer (GPC3_Apt_), an “H-rGO-Pd NPs-GPC3_Apt_/GPC3/GPC3_Ab_” sandwich complex was formed with peroxidase-like properties which enhanced H_2_O_2_ to reduce the silver (Ag) ions in solution to metallic Ag, resulting in the deposition of silver nanoparticles (Ag NPs) on the surface of the biosensor. The amount of deposited Ag, which was derived from the amount of GPC3, was quantified by the differential pulse voltammetry (DPV) method. Under ideal circumstances, the response value was linearly correlated with GPC3 concentration at 10.0–100.0 μg/mL with R^2^ of 0.9715. When the GPC3 concentration was in the range from 0.01 to 10.0 μg/mL, the response value was logarithmically linear with the GPC3 concentration with R^2^ of 0.9941. The limit of detection was 3.30 ng/mL at a signal-to-noise ratio of three and the sensitivity was 1.535 μAμM^−1^cm^−2^. Furthermore, the electrochemical biosensor detected the GPC3 level in actual serum samples with good recoveries (103.78–106.52%) and satisfactory relative standard deviations (RSDs) (1.89–8.81%), which confirmed the applicability of the sensor in practical applications. This study provides a new analytical method for measuring the level of GPC3 in the early diagnosis of HCC.

## 1. Introduction

Globally, hepatocellular carcinoma (HCC) has been evaluated as one of the most common malignant malignancies with high prevalence and fatality rates [1]. The survival rate of HCC patients has been suggested to increase with the earlier detection of this malignancy [2]. The existing imaging methods, including ultrasound (US), magnetic resonance imaging (MRI), computed tomography (CT), and photoacoustic imaging (PAI), find difficulty in detecting HCC, and all these detection methods require expensive instruments as well as specialized personnel. Therefore, the development of HCC diagnosis is crucial to increase the chance of effective treatment and reduce HCC-related mortality [3].

Immunoassay detection of serum biomarkers including enzyme-linked immunoassay (ELISA) and bioluminescence enzyme immunoassay (BLIEA) has been widely used in clinical practice for the diagnosis of HCC [4,5,6]. Glypican-3 (GPC3), as an emerging biomarker, has been shown to be beneficial for early clinical screening of HCC [7,8]. A study showed that the serum concentration of GPC3 (108.67 ± 230.04 ng/mL) in HCC patients was much higher than those in healthy persons (3.99 ± 7.68 ng/mL) [9]. Therefore, the use of GPC3 as one of the detection criteria for HCC will potentially improve the detection rate of HCC. 

ELISA serum kits are widely used to detect antigens or antibodies in clinical practice by using double antibodies and labeled-horseradish peroxidase (HRP) to form sandwich-type structures [10]. On the one hand, antibodies have strong specificity in recognizing the target protein. On the other hand, the sandwich structure provides a more stable result for HRP color development. It is worth mentioning that the advent of nucleic acid aptamer complements the disadvantages that antibodies are not easy to store and has the advantage of antibody specificity [11,12]. Compared with natural enzymes such as HRP, artificial nanozymes have the advantages of high catalytic efficiency, high stability, and low price [13,14].

In recent years, electrochemical detection has alternatively been used as a powerful technique for many point-of-care (POC) sensors due to its inherent advantages of low cost, portability, and fast response [15,16]. For instance, Zhou et al. designed a dual-recognition sandwich electrochemical biosensor for circulating tumor (CTC) detection based on PdPtCuRu nanospheres with catalytic hydrogen peroxide properties to improve the specificity, and the constructed sandwich biosensor exhibited good specificity and accuracy in spiked serum samples [17]. A study showed that sandwich structure greatly improves the stability, sensitivity, and specificity of the sensor to detect the target protein [18]. Therefore, the construction of a sandwich electrochemical biosensor could provide new opportunities to improve the sensitivity for HCC detection.

Nanozymes are simulated enzymes composed of nanomaterials, which have two characteristics of nanomaterials and enzyme-like activities. Nanozymes can solve the shortcomings of the natural enzyme such as high cost and variability, but the activity is slightly lower than that of the natural enzyme. Nanozymes and reaction substrates are accompanied by electron transfer and valence changes, showing REDOX enzyme activities. Compared with ordinary bio-signal, the electrochemical signal generated by nanozyme-catalyzed amplification technique can be enhanced by the reaction of the chromogenic substrate [19,20]. Among them, peroxidase-like catalytic silver deposition has been an effective way for signal enhancement due to high catalytic activity, in situ highly efficient enzymatic catalysis of Ag NPs deposition, and increased conductivity resulting from Ag deposition [21]. In another aspect, Hemin, an important natural porphyrin iron compound, is often used as a catalyst to replace natural enzymes due to its good peroxidase properties [22]. Similarly, palladium nanoparticles (Pd NPs) can be used not only as a highly stable catalase but also as a conductivity material to improve electrical conductivity [23]. Reduced graphene oxide (rGO), a two-dimensional structure of carbon materials, is a useful carrier with strong electrical conductivity and high biocompatibility due to its large specific surface area [24].

Given the above considerations, our study aimed to generate a novel sandwich-structure electrochemical biosensor for GPC3 detection based on hemin-reduced graphene oxide-palladium nanoparticles (H-rGO-Pd NPs) nanozyme peroxidase-like catalytic silver deposition for signal amplification, combining GPC3 aptamer (GPC3_Apt_) and GPC3 antibody (GPC3_Ab_) as recognition elements. GPC3_Apt_ was labelled on the binding sites of H-rGO-Pd NPs nanozyme through π-π action and Pd-N coordination interaction. The H-rGO-Pd NPs-GPC3_Apt_ signal probe not only improves the electron transfer rate but also enhances the number of fixed biomolecules. GPC3_Ab_, as a capture probe, was adsorbed on the surface of a Au NPs@rGO-modified screen-printed electrode (SPE). In the presence of GPC3, an “H-rGO-Pd NPs-GPC3_Apt_-GPC3-GPC3_Ab_” sandwich-structure complex with peroxide properties was formed by specific binding, and further catalyzed the reaction of H_2_O_2_ with AgNO_3_ to deposit Ag NPs on the sensor surface. The dissolution current of Ag NPs could be measured using differential pulse voltammetry (DPV). The analytical performance in terms of working curve, linear range, sensitivity, specificity, reproducibility, and stability of the proposed GPC3 electrochemical nanobiosensor was discussed. 

## 2. Results and Discussion

### 2.1. The Analysis Principle and Feasibility of GPC3 Electrochemical Aptasensor

**Figure 1:**
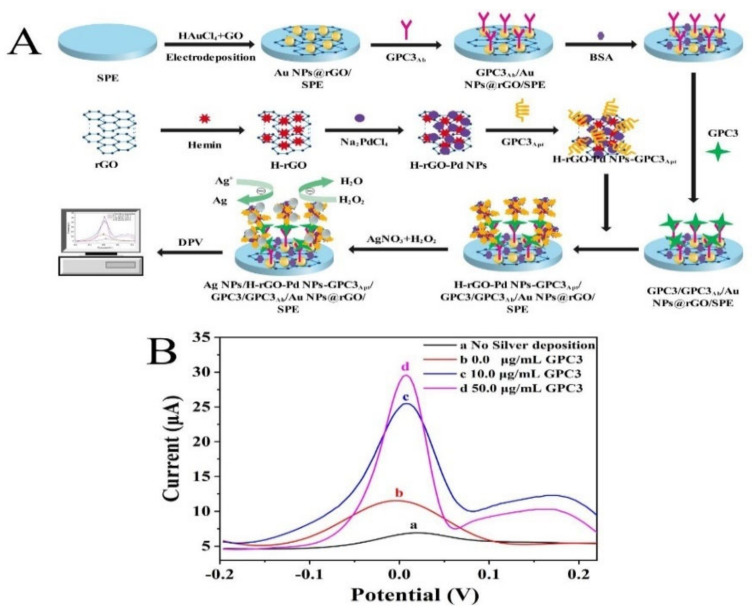
(**A**) Schematic principle of the GPC3 electrochemical nanobiosensor based on H-rGO-Pd NPs nanozyme. (**B**) DPV curves for the feasibility of the GPC3 electrochemical nanobiosensor with GPC3 or without GPC3.

Figure 1A illustrates the detection principle of the GPC3 electrochemical nanobiosensor based on H-rGO-Pd NPs nanozyme peroxidase-like catalytic silver deposition for signal amplification, combining the GPC3 aptamer (GPC3_Apt_) and the GPC3 antibody (GPC3_Ab_) as recognition elements. Firstly, the H-rGO-Pd NPs nanozyme with good peroxidase-like catalytic properties was prepared by a two-step reduction method, and the H-rGO-Pd NPs-GPC3_Apt_ detection probe was prepared through π-π action, and Pd-N coordination interaction. Then, the Au NPs@rGO was modified on the surface of pretreated SPE by electrodeposition, leading to the formation of Au NPs@rGO/SPE. After that, GPC3_Ab_ was immobilized on Au NPs@rGO/SPE by Au-N bonding as well as physical adsorption. When the GPC3 was added, the specific recognition reaction between the GPC3_Ab_ and GPC3 produced an antibody–antigen complex and arranged on the electrode surface. Next, the H-rGO-Pd NPs-GPC3_Apt_ detection probe was fixed on the electrode surface by π-π bond and electrostatic adsorption. Both GPC3_Ab_ and H-rGO-Pd NPs-GPC3_Apt_ specifically bonded with GPC3 to form the H-rGO-Pd NPs-GPC3_Apt_/GPC3/GPC3_Ab_ sandwich-structure complex with a stable spatial structure as well as catalytic performance, which could induce the reduction in the Ag ions in the solution containing H_2_O_2_ and AgNO_3_ solution for the deposition of Ag NPs on the surface of Au NPs@rGO/SPE. The metallic Ag NPs deposited on Au NPs@rGO/SPE could produce detectable anodic stripping signals, which can be determined by DPV. Since the amount of H-rGO-Pd NPs-GPC3_Apt_/GPC3/GPC3_Ab_ affects the Ag NPs deposition which further leads to the change of the sensor response current, the standard curve was determined by studying the relationship between the sensor response current and GPC3 concentration.

Herein, H-rGO-Pd NPs revealed good conductivity, nontoxicity, and high peroxidase-like catalytic properties because of the peroxidase properties of Hemin, good catalyst-supporting material of rGO, and the efficient catalytic synergies of Pt NPs.

By using the DPV method, the feasibility of the GPC3 electrochemical nanobiosensor was determined under the potential voltage range of (−0.2–0.4 V) (Figure 1B). In the absence of Ag (i.e., no silver deposition, curve a), there was an insignificant response current, indicating that the determination of GPC3 is achieved by the anodic stripping peak current of the depositing Ag. In the absence of GPC3 protein (curve b), the response current was slightly higher than that of the curve a. This reason for this phenomenon was that H_2_O_2_ was able to react with AgNO_3_ slowly without catalytic substances, which left a small amount of deposited Ag on the electrode surface. Under the catalysis of H-rGO-Pd NPs peroxidase-like activity, the GPC3 nanobiosensor detected a significant current response (curve c and curve d). Moreover, the current response significantly increased when the concentration of GPC3 went up from 10.0 to 50.0 μg/mL. It was positively correlated with GPC3 concentrations. The feasibility analysis suggested that the “H-rGO-Pd NPs-GPC3_Apt_/GPC3/GPC3_Ab_” sandwich complex can effectively catalyze the reaction of H_2_O_2_ and AgNO_3_ on the surface of electrodes. Being heavily coated with Ag NPs amplified the current signal of the sensor, further indicating that the electrochemical nanobiosensor was capable of detecting GPC3.

### 2.2. Characterization of H-rGO-Pd NPs

**Figure 2:**
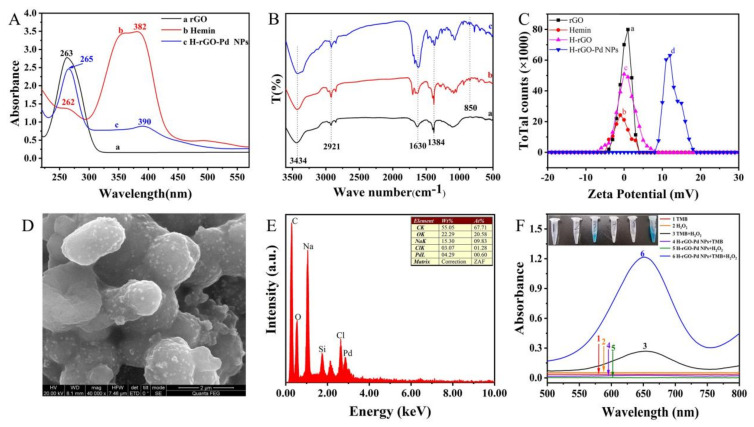
(**A**) UV-vis spectrum of H-rGO-Pd NPs. (**B**) FT-IR characterization of H-rGO-Pd NPs. (**C**) Zeta potential analysis diagram of H-rGO-Pd NPs. (**D**) SEM image of H-rGO-Pd NPs. (**E**) EDS image of H-rGO-Pd NPs. (**F**) The peroxidase-like activities of H-rGO-Pd NPs.

The UV-vis spectra of rGO, hemin, and H-rGO-Pd NPs were shown in Figure 2A. The peak of 263 nm was the strongest absorption peak of rGO (curve a), indicating that rGO was successfully reduced [25]. The peak of 382 nm was the Soret peak of hemin (curve b). H-rGO-Pd NPs (curve c) had similar absorption peaks at 265 nm and 390 nm, which was consistent with rGO and hemin, and the absence of a characteristic absorption peak for the reduction of Pd ions to zero-valent Pd NPs [26], which indicated that H-rGO-Pd NPs may be successfully synthesized.

FT-IR spectra of rGO (curve a), hemin (curve b), and H-rGO-Pd NPs (curve c) were shown in Figure 2B. All of them had the characteristic absorption peaks of O-H at 3434 cm^−1^, a constriction vibration peak of C-H at 2918 cm^−1^, a vibration peak of C=O at 1630 cm^−1^, and a vibration peak of C-O at 1384 cm^−1^. H-rGO-Pd NPs and hemin have a common asymmetric stretching peak at 850 cm^−1^ which is from the formation of Fe-O bond in hemin [27]. Moreover, compared with the strong intensity of the peak of hemin at 1384 cm^−1^ (C-O), the weak intensity of H-rGO-Pd NPs can be described by the binding interaction between H- rGO and the Pd NPs, indicating the H-rGO-Pd NPs may have been successfully synthesized.

Characterization of the H-rGO-Pd NPs was performed using the Zeta particle size analyzer. As shown in Figure 2C, the Zeta potential of rGO (curve a), hemin (curve b), H-rGO (curve c), and H-rGO-Pd NPs (curve d) were 1 mV, −1 mV, 0 mV, 12 mV, respectively. The H-rGO-Pd NPs were shifted to positive potential due to the presence of a small amount of Pd^2+^ in the solution. It can be seen that H-rGO-Pd NPs were successfully prepared.

By using SEM, the surface morphology of H-rGO-Pd NPs was characterized. As shown in Figure 2D, many dark gray particles with relatively uniform particles and a folded film-like structure appeared on the surface of H-rGO-Pd NPs, indicating that the Pd NPs were successfully attached to H-rGO, i.e., the H-rGO-Pd NPs had been successfully prepared. In addition, the SEM characterization diagram of rGO and H-rGO is shown in Figure S1. Meanwhile, the EDS energy spectrum of H-rGO-Pd NPs was measured on silicon wafers. As shown in Figure 2E, the H-rGO-Pd NPs were rich in C and O elements and Na elements and contained a small amount of Pd elements and Cl elements. Furthermore, the TEM was also used to characterize the H-rGO-Pd NPs (see in Figure S1C).

The peroxidase-like characteristics of H-rGO-Pd NPs were verified in Figure 2F. Both the TMB and H_2_O_2_ were colorless and there was no absorption peak (curves 1, 2). After mixing TMB and H_2_O_2_, a weak reaction occurred, producing the mixed solution with a light blue color (insert figure in Figure 2E) and a soft absorption peak (curve 3). The results indicated that a few hydroxyl radicals in H_2_O_2_ could oxidize the TMB. The H-rGO-Pd NPs solution was added to two solutions containing TMB (curve 4) or H_2_O_2_ (curve 5), respectively; however, no change was found in both solutions. When all TMB, H_2_O_2_, and H-rGO-Pd NPs were mixed, the mixture turned dark blue from being colorless (Figure 2F). Moreover, the absorption peak (curve 6) was much higher than that of the mixed solution of TMB and H_2_O_2_ without H-rGO-Pd NPs. This indicated that H-rGO-Pd NPs can effectively catalyze the reaction between H_2_O_2_ and TMB, and thus have peroxidase activity. 

Furthermore, to explore whether the combination of GPC3_Apt_ and H-rGO-Pd NPs was successful, the H-rGO-Pd NPs-GPC3_Apt_ was characterized and analyzed by UV-vis (see Figure S2).

### 2.3. Electrochemical Study of Au NPs@rGO/SPE and Raman Spectra of the Modified Electrodes

**Figure 3:**
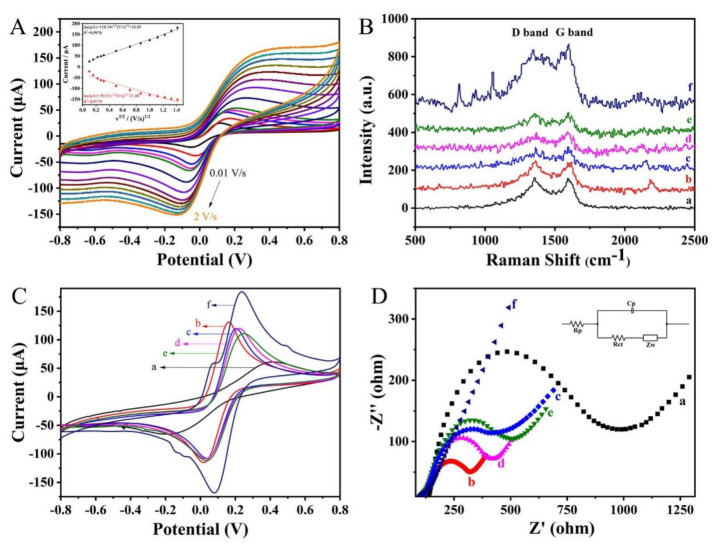
(**A**) CV characterization of Au NPs@rGO/SPE in 5.0 mM [Fe(CN)_6_]^3−/4−^ and 0.1 M KCl solution at 0.01 V/s, 0.025 V/s, 0.05V/s, 0.075 V/s, 0.1 V/s, 0.5 V/s, 0.75 V/s, 1 V/s, 1.25 V/s, 1.5 V/s, and 2 V/s. (**B**) Raman spectra of electrode for different processing steps. (**C**) CV characterization. (**D**) EIS characterization of the electrochemical nanosensor preparation process in 5.0 mM [Fe(CN)_6_]^3−/4−^ and 0.1 M KCl solution at the frequency range from 0.1 Hz to 10 kHz. (a, SPE; b, Au NPs@rGO/SPE; c, GPC3_Ab_/Au NPs@rGO/SPE; d, GPC3/GPC3_Ab_/Au NPs@rGO/SPE; e, H-rGO-Pd NPs-GPC3_Apt_/GPC3/GPC3_Ab_/AuNPs@rGO/SPE; f, AgNPs/H-rGO-PdNPs-GPC3_Apt_/GPC3/GPC3_Ab_/Au NPs@rGO/SPE).

As displayed in Figure 3A, a different scan rate (0.01–2 V/s) was performed on Au NPs@rGO/SPE in a solution containing 1 M KCl and 5 mM [Fe(CN)_6_]^3−/4−^ by using the CV method. When the scan rate increased, the peak of the CV curve gradually increased. The relevant linear regression equations were as follows (in Figure 3A): where the anode: Ipa (μA) = 110.34 v^1/2^+16.69 (R^2^ = 0.9976), and the cathode: Ipc (μA) = −92.51 v^1/2^–31.88 (R^2^ = 0.9779), indicating that the redox process on Au NPs@rGO/SPE was a diffusion-controlled reaction [28].

The Raman microscopy of each modified GPC3 electrochemical nanosensor is shown in Figure 3B. The G-band reflected the order and integrity of materials containing carbon, and the D-band indicated the degree of defect in the C atomic lattice [29]. The I_D_/I_G_ ratio was 1.05 for the Raman curve of SPE (curve a). Curve b was the Raman curve of Au NPs@rGO/SPE with an I_D_/I_G_ of 1.04. The Raman I_D_/I_G_ was 1.02 for GPC3_Ab_/Au NPs@rGO/SPE (curve c). The Raman curves d and e were GPC3/GPC3_Ab_/Au NPs@rGO/SPE and H-rGO-Pd NPs-GPC3_Apt_/GPC3/GPC3_Ab_/Au NPs@rGO/SPE, with I_D_/I_G_ of 0.95 and 0.98, respectively. Curve f was the sensor electrode where Ag NPs were deposited, and its I_D_/I_G_ was 0.84, showing that the deposition of a large number of Ag NPs had filled the defects inside the composite on the electrode surface.

### 2.4. Electrochemical Characterization of the Modified Electrodes

Cyclic voltammetry (CV) was performed to show the adsorption state of the electrode at different stages (Figure 3C) in a PBS solution (0.1 M, pH 7.0) containing 5.0 mM K_3_Fe(CN)_6_/K_4_Fe(CN)_6_ and 0.1 M KCl solution with a voltage range of −0.8 to 0.8 V and a scanning speed of 50 mV/s. The activated bare SPE (Ip: 61.1 μA, curve a) exhibited the lowest redox peaks. The Au NPs@rGO/SPE (Ip: 130.7 μA, curve b) increased its redox peak. The redox peak of GPC3_Ab_/Au NPs@rGO/SPE (Ip: 119.8 μA, curve c) decreased slightly due to the presence of GPC3_Ab_, which hindered the electron transport. In particular, the interaction between GPC3 and GPC3_Ab_ hindered electron transfer, and the redox peak of GPC3/GPC3_Ab_/Au NPs@rGO/SPE was slightly changed (Ip: 119.5 μA, curve d). When H-rGO-Pd NPs-GPC3_Apt_ incubated on the electrode (Ip: 110.8 μA, curve e), the current failed to rise but decreased slightly because of the current hindered by the incubation of the aptamer, to some extent. When Ag NPs continued to be deposited on the surface of the electrode due to the catalytic action of H-rGO-Pd NPs peroxidase-like activity, the current of Ag NPs/H-rGO-Pd NPs-GPC3_Apt_/GPC3_Ab_/Au NPs@rGO/SPE (Ip: 183.9 μA, curve f) increased significantly.

Furthermore, the surface area was determined by utilizing the CV result of [Fe(CN)_6_]^3−/4−^, derived from Randles–Sevcik’s formula [30,31,32].

(1) $$I_{P}=2.69\times 10^{5}AD^{1/2}n^{3/2}v^{1/2}C$$

In this formula, *Ip* represents the current peak (μA), *A* represents the sensor’s effective surface area (cm^2^), *D* is the medium proliferation parameter [Fe(CN)_6_]^3−/4−^ (6.70 × 10^−6^ cm^2^ s^−1^), *n* is the number of electrons involved ([Fe(CN)_6_]^3−/4−^, n = 1), *v* represents the scanning rate (0.1 V/s), and *C* represents the concentration of redox medium (5 mM/L). The surface area of different electrodes was calculated in square measure and placed in the following order: SPE (0.0555 cm^2^) < H-rGO-Pd NPs-GPC3_Apt_/GPC3/GPC3_Ab_/Au NPs@rGO/SPE (0.1006 cm^2^) < GPC3/GPC3_Ab_/Au NPs@rGO/SPE (0.1085 cm^2^) < GPC3_Ab_/Au NPs@rGO/SPE (0.1088 cm^2^) < Au NPs@rGO/SPE (0.1187 cm^2^) < Ag NPs/H-rGO-Pd NPs-GPC3_Apt_/GPC3/GPC3_Ab_/Au NPs@rGO/SPE (0.1670 cm^2^). These results confirmed that both H-rGO-Pd NPs nanozyme-catalyzed silver deposition and SPE deposition of Au NPs@rGO significantly increased the conductivity of the electrode, further facilitating electron transfer. 

EIS could be an effective tool for characterizing the properties of electron switches in a range of electrode modifications. Thus, 5 mM of [Fe(CN)_6_]^3−/4−^ solution mixed with 1 M of KCl as the electrolyte solution, every electrode in the sensor construction process underwent an EIS scan at a constant voltage of 5 mV. As shown in Figure 3D, the impedance value of the SPE was 991 Ω (curve a). After the electrodeposition of Au NPs@rGO, its impedance also dropped sharply (320 Ω, curve b). Moreover, the presence of GPC3_Ab_ hindered electron transfer, and the impedance became larger (414 Ω, curve c). After GPC3 was incubated, the binding of GPC3 and GPC3_Ab_ hindered electron transfer (423 Ω, curve d), H-rGO-Pd NPs-GPC3_Apt_ was dropped on the electrode, the impedance increased (504 Ω, curve e) because of the presence of the aptamer hindering the electron transfer. Finally, after silver particles were deposited on the surface of the sensor, the electrode impedance decreased sharply (132 Ω, curve f), indicating that H-rGO-Pd NPs effectively catalyzed the reaction of H_2_O_2_ and AgNO_3_. It was concluded that the electrode impedance was significantly reduced by depositing a large amount of Ag NPs on its surface.

### 2.5. SEM Spectroscopy Characterization of the Modified Electrodes

**Figure 4:**
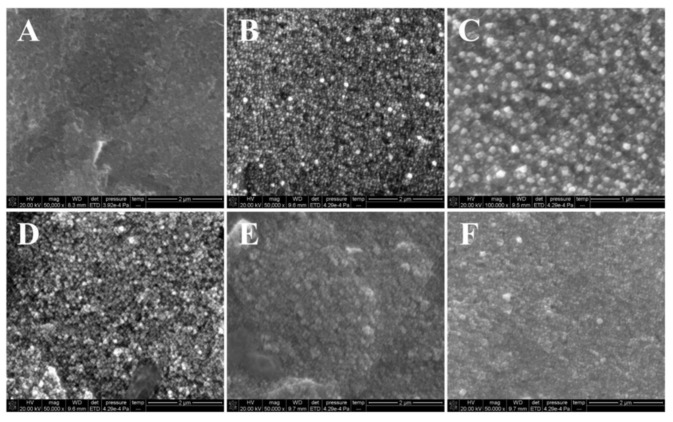
SEM images of the (**A**) bare SPE, (**B**) Au NPs@rGO/SPE, (**C**) GPC3_Ab_/Au NPs@rGO/SPE, (**D**) GPC3/GPC3_Ab_/Au NPs@rGO/SPE, (**E**) H-rGO-Pd NPs-GPC3_Apt_/GPC3/GPC3_Ab_/Au NPs@rGO/SPE, and (**F**) Ag NPs/H-rGO-Pd NPs-GPC3_Apt_/GPC3/GPC3_Ab_/Au NPs@rGO/SPE.

The GPC3 electrochemical nanosensor was characterized by using SEM. The surface of the bare SPE (Figure 4A) was relatively flat. When Au NPs@rGO were deposited on SPE, there were two different uniform particles presented on the surface of Au NPs@rGO/SPE (Figure 4B), one was darker in color and the other was brighter. After modifying GPC3_Ab_, the GPC3_Ab_/Au NPs@rGO/SPE (Figure 4C) showed a white film. When GPC3 was present, the formation of GPC3/GPC3_Ab_/Au NPs@rGO/SPE (Figure 4D) found white spheroids on the surface of the electrode. After the H-rGO-Pd NPs-GPC3_Apt_ was fixed, the surface of the H-rGO-Pd NPs-GPC3_Apt_/GPC3/GPC3_Ab_/Au NPs@rGO/SPE (Figure 4E) became flat, and a folded film was seen. On the surface of the Ag NPs/H-rGO-Pd NPs-GPC3_Apt_/GPC3/GPC3_Ab_/Au NPs@rGO/SPE (Figure 4F), Ag NPs were deposited, and shiny silver particles were found. The above results suggested that Au NPs@rGO, GPC3_Ab_, GPC3, and H-rGO-Pd NPs-GPC3_Apt_ electrode surfaces were modified.

### 2.6. Optimization of Conditions for GPC3 Electrochemical Aptasensor

To improve the performance of the aptasensor, some experimental conditions including the concentration of the GPC3_Apt_, incubation temperature, incubation time, and the amount of H-rGO-Pd NPs nanozymes were optimized. As shown in Figure S3, 5 μmol/L of GPC3_Apt_, 25 °C of incubation temperature, 60 min of incubation time, and 4 μL of H-rGO-Pd NPs nanozymes were chosen as the optimized detection conditions for subsequent experiments.

### 2.7. Analytical Performance of GPC3 Electrochemical Aptasensor

**Figure 5:**
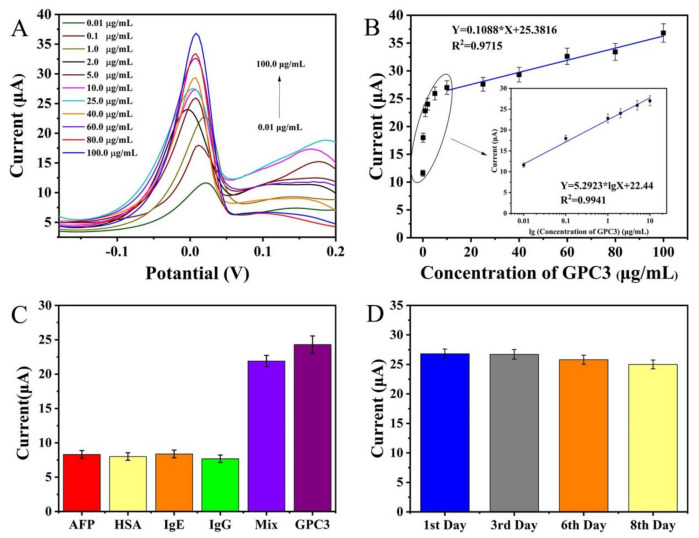
(**A**) DPV curves of the electrochemical nanobiosensor with different GPC3 concentrations from 0.01 to 100.0 μg/mL in the potential range of −0.2 to 0.4 V at a scan rate of 100 mV s^−1^. (**B**) Calibration plot of the GPC3 electrochemical nanobiosensor in two different concentration ranges of 10.0–100.0 μg/mL and 0.01–10.0 μg/mL. (**C**) Histogram for the specificity investigation of the proposed GPC3 electrochemical nanobiosensor (the concentration of GPC3 was 1.0 μg/mL and the concentration of each interfering agent was 10.0 μg/mL). (**D**) Histogram for the stability analysis of the proposed GPC3 electrochemical nanobiosensor (the concentration of GPC3 was 1.0 μg/mL). All the above-mentioned values are presented as the median from the analysis of three independent experiments and the error bars indicate the relative standard deviation.

Herein, the GPC3_Ab_-GPC3-H-rGO-Pd NPs-GPC3_Apt_ sandwich nanobiosensor was constructed using H-rGO-Pd NPs-GPC3_Apt_ as the signal probe and GPC3_Ab_ as the capture probe. In the presence of H-rGO-Pd NPs with peroxidase-like catalytic properties, H_2_O_2_ could reduce the Ag ions in the solution to metallic Ag, which was deposited on the surface of the electrode. The ultrasensitive detection of GPC3 was achieved by the DPV method for the anodic stripping signal of the deposited Ag NPs. Under optimal conditions, the DPV curves of the electrochemical nanobiosensor with different GPC3 concentrations were obtained and shown in Figure 5A. When the GPC3 concentration increased from 0.01 to 100.0 μg/mL, the response current of the nanobiosensor increased. This was due to H-rGO-Pd NPs-GPC3_Apt_ specifically recognizing more GPC3 to form more sandwich complexes, resulting in increasing metallic Ag NPs deposition and enhancing electrochemical signal responsiveness. The electrochemical nanobiosensor working curve was shown in Figure 5B. The electrochemical nanobiosensor responded logarithmically linearly to the GPC3 concentration from 0.01 to 10.0 μg/mL. The linear regression equation was Y = 5.2923 × lgX + 22.44 (Y represented the current response, X represented the concentration of GPC3) with R^2^ of 0.9941. When the GPC3 concentration was from 10.0 to 100.0 μg/mL, the electrochemical nanobiosensor responded linearly to GPC3 concentrations. This equation corresponded to Y = 0.1088 × X + 25.3816 in linear regression with R^2^ of 0.9715. The response time of the nanobiosensor was 30 min, and the sensitivity of the nanobiosensor was calculated to be 1.535 μA μM^−1^ cm^−2^, which was calculated by dividing the calibration curve gradient by the electrode’s valid area [33]. The detection limit of detection (LOD) for the nanobiosensor was evaluated using the signal-to-noise ratio (S/N = 3), and the LOD of 3.30 ng/mL was obtained [34].

The comparisons between the developed electrochemical nanobiosensor and other GPC3 detection methods are shown in Table 1. Compared with other methods, the GPC3 nanobiosensor had a wider detection range and higher sensitivity. This may be due to the signal amplification strategy of silver deposition catalyzed by the enzyme being used to strengthen the response current signal to a certain extent, resulting in higher sensitivity. However, the LOD value obtained by this project (3.30 ng/mL) was close to that of the previous research group (2.86 ng/mL) [35], but was slightly worse than those of ELISA, BLEIA, or TRFIA [4,10,36]. On the one hand, the impedance of the “antibody–antigen–aptamer” sandwich complex was larger, resulting in slightly poor LOD. On the other hand, the “antibody-–antigen–aptamer” sandwich complex exhibited spatial stereoscopic properties, which would obstruct the catalytic deposition of Ag NPs by H-rGO-Pd NPs nanozymes, to some extent, resulting in a less current change. Although the LOD value of the designed sensor was slightly higher, the antibody–antigen–aptamer sandwich structure and the enzyme-catalyzed silver deposition signal amplification strategy improved the stability and sensitivity of the sensor. Moreover, due to its easy synthesis and lower cost, the GPC3 nanobiosensor can be an ideal solution for designing high-sensitivity clinical tests.

### 2.8. Specificity, Stability, and Reproducibility of GPC3 Electrochemical Nanobiosensor

AFP, HSA, IgE, and IgG were chosen as interfering substances to examine the specificity of the electrochemical nanobiosensor. Briefly, 1.0 μg/mL GPC3, one of the above four interfering chemicals (10.0 μg/mL), or a mixture (all interfering substances) was mixed with GPC3 in a 1:1 ratio with the same concentration (1.0 μg/mL), for which the results are shown in Figure 5C. According to Figure 5C, the response current value of GPC3 was 24.30 μA, which was about three times that of the current value of other interfering substances (AFP, 8.31 μA; HSA, 8.01 μA; IgE, 8.38 μA; IgG, 7.68 μA). In addition, the response of the mixture (21.90 μA) was very close to that of GPC3. Therefore, the presence of other interfering substances had little influence on the corresponding current value of the nanobiosensor, indicating that the specificity of the nanobiosensor would meet the detection requirements.

To detect the GPC3 protein (1.0 µg/mL) and analyze its stability by using the DPV technique, the GPC3 electrochemical nanobiosensor was kept at 4 °C in a refrigerator, and would be taken out at various intervals (1, 3, 6, and 8 days) (Figure 5D). The results showed that the response current of nanobiosensor decreased to 99.5%, 96.0%, and 93.1% of the original current value after 3, 6, and 8 days, respectively, indicating the GPC3 electrochemical nanobiosensor had good short-term stability. Furthermore, the reproducibility of the GPC3 electrochemical nanobiosensor was also studied (see Figure S4). The relative standard deviations (RSDs) of the response currents of five sensors was 2.56%, suggesting that the GPC3 electrochemical nanobiosensor can be reproducible.

### 2.9. Analysis of GPC3 in Actual Human Serum Samples

To verify that the nanobiosensor would be applied in actual serum detection, GPC3 in human serum samples was detected by standard addition methods under optimal conditions. The approval was first received from the Ethics Committee of the Guangxi Key Laboratory of Metabolic Disease Research of the 924th Hospital of the Chinese People’s Liberation Army Joint Logistic Support Force (Guilin, China). Three kinds of serum samples were prepared and detected with the same process instead of GPC3 standard solution and the results are shown in Table 2. As seen in Table 2, the recovery rate ranged from 103.78 to 106.52%. There was a range of 1.89% to 8.81% in the RSDs value (n = 3). The developed sensor showed a promising ability for the determination of GPC3 in actual serum samples.

## 3. Materials and Methods

### 3.1. Chemicals and Reagents

Graphene oxide (GO) was obtained from Xianfeng Nano Co., Ltd. (Nanjing, China). Hemin, GPC3, Sodium tetrachloropalladate (Na_2_PdCl_4_), chloroauric acid (HAuCl_4_·4H_2_O), silver nitrate (AgNO_3_), Sodium chloride (NaCl), hydrogen peroxide (H_2_O_2_), ascorbic acid (AA), and ethylene glycol (EG) were obtained from Xilong Scientific Co., Ltd. (Shantou, China). Human serum albumin (HSA), Sodium hydroxide (NaOH), and bovine serum albumin (BSA) were obtained from Macklin Biochemical Co., Ltd. (Shanghai, China). GPC3 antibody (GPC3_Ab_) and GPC3 aptamer (GPC3_Apt_, 5’-NH_2_-TAA CGC TGA CCT TAG CTG GAT TTT ACA TGT TCC A-3’) [27], Polydiallyldimethylammonium chloride (PDDA), and 3,3’,5,5’-Tetramethylbenzidine (TMB) were purchased from Sigma-Aldrich Trading Co., Ltd. (Shanghai, China). Phosphate buffer solution (PBS, 0.1 mol/L) was used as the electrolyte in the measuring system.

### 3.2. Electrochemical Measurements and Apparatus

All electrochemical experiments were conducted on the electrochemical workstation (CHI660E, Shanghai Chenhua Instrument Co., Ltd., Shanghai, China) at room temperature. Electrochemical measurements were performed with conventional screen-printed electrodes (SPE, PK215, Nanjing Yunyou Biotechnology Co., Ltd., Nanjing, China), one of the carbon paste electrodes served as an auxiliary electrode, the other as a working electrode (surface area = 0.07 cm^2^, Φ = 3 mm), and the reference electrode was Ag/AgCl inert.

Transmission electron microscopy (TEM) was performed with a JEM-2100F electron microscope (JEM-2100F, Nippon Electronics Co., Tokyo, Japan) at 100 kV accelerating voltage. A Quanta 200 Fifield scanning electron microscope (QUANTA 200, FEI COMPANY, Hillsboro, USA) was used for scanning electron microscopy (SEM). It was measured at 400–4000 cm^−1^ using the Fourier Transform Infrared spectrometer (FT-IR, Nicolet IS10, Nicolet, Waltham, USA). A DXR Raman microscope (DXR3, Thermo-Fisher Scientific, Waltham, USA) was used to measure Raman spectra between 200 and 3500 cm^−1^. The wavelength range of the ultraviolet-visible spectroscopy (UV-vis) (UH5300, HITACHI, Tokyo, Japan) was 200–600 nm.

### 3.3. Preparation of the H-rGO-Pd NPs Nanozyme and H-rGO-Pd NPs-GPC3_Apt_ Detection Probe

H-rGO-Pd NPs nanozymes were prepared by a two-step reduction method. First, well-dispersed hemin-reduced graphene oxide (H-rGO) solution was prepared as described in our earlier study [35]. Briefly, 10.0 mg GO powder was dispersed in 20.0 mL pure water, and crushed by cell crusher ultrasonic for 2 h to obtain GO solution (0.5 mg/mL), then Hemin aqueous solution (0.5 mg/mL) was added to mix evenly, and 30 μL ammonia solution (NH_3_·H_2_O) and 100.0 μL hydrazine hydrate (NH_2_-NH_2_) were added. After mixing, the solution was centrifugally cleaned in a water bath at 60 °C for 4 h to obtain the H-rGO solution. Second, 2.0 mL of PDDA (ω = 0.2%) and 5.0 mL of NaCl (0.2 M) were added into the 10.0 mL of H-rGO solution (0.5 mg/mL) and stirred for 12 h to form PDDA-modified H-rGO solution. Then, 2.0 mL of Na_2_PdCl_4_ (20 mM) and 10.0 mL of EG were added into the PDDA-modified H-rGO solution. After being stirred overnight, the pH of the solution was adjusted to 12 with 1.0 M of NaOH. Furthermore, the above solution was refluxed for 4 h at 140 °C. Finally, the H-rGO-Pd NPs were obtained by centrifuging and drying.

H-rGO-Pd NPs-GPC3_Apt_ detection probe was prepared through π-π action and Pd-N coordination interaction. A total of 100.0 μL of GPC3_Apt_ solution (5 μM) and 200.0 μL of H-rGO-Pd NPs (0.5 mg/mL) were sonically mixed overnight at room temperature. Then, the solution was centrifuged at 12,000 rpm for 20 min to remove free aptamers. Thereafter, the H-rGO-Pd NPs-GPC3_Apt_ detection probe (1.0 mg/mL) was obtained after the residue was dispersed in Tris-EDTA buffer.

### 3.4. Construction of the GPC3 Electrochemical Nanobiosensor

Firstly, the SPE was immersed in 5 mL of H_2_SO_4_ solution (0.5 M) and activated by an electrochemical cyclic scanning method with a scanning speed of 0.5 V/s and a scanning voltage between 0.4 and 1.2 V for 20 cycles. Secondly, the activated SPE was placed in a 5 mL mixed aqueous solution of HAuCl_4_ (2.5 mL, ω = 0.05%) solution and GO solution (2.5 mL, 1.0 mg/mL), and electrodeposited under cyclic voltammetry (CV) strategy for 120 s under magnetic stirring in the voltage range of −0.5–1.0 V. The scanning rate was 0.4 V/s and the scanning period was 10 cycles. After electrodeposition, the SPE was rinsed with water several times and dried to get Au NPs@rGO/SPE [38,39,40,41]. Thirdly, 1.0 μL GPC3_Ab_ was dropped on the surface of Au NPs@rGO/SPE electrode and incubated for 30 min at 25 °C. Lastly, 6.0 μL of 1% BSA solution was added dropwise to GPC3_Ab_/AuNPs@rGO/SPE and incubated for 30 min at 25 °C to block non-specific active sites [42], After each step, ultrapure water was used to clean the electrodes and to dry them.

### 3.5. GPC3 Detection Based on H-rGO-Pd NPs Nanozymes-Catalyzed Silver Deposition

Firstly, 1.0 μL GPC3 standard solution (different concentration) was added dropwise onto the GPC3_Ab_/AuNPs@rGO/SPE surface and incubated at 25 °C for 30 min. Secondly, 4.0 μL of H-rGO-Pd NPs-GPC3_Apt_ (1.0 mg/mL) solution was added dropwise onto the surface of GPC3/GPC3_Ab_/AuNPs@rGO/SPE and incubated at 25 °C for 60 min. Thirdly, 6.0 μL of H_2_O_2_ (100 mmol/L) and 3.0 μL of AgNO_3_ (6.0 mmol/L) solution were added dropwise onto the surface of H-rGO-Pd NPs-GPC3_Apt_/GPC3/GPC3_Ab_/Au NPs@rGO/SPE and kept in the dark at 25 °C for 30 min. The electrode was rinsed three times with water. Lastly, the electrode was inserted into a 4.0 mL glycine-NaOH buffer solution (0.05 M, pH 8.5) containing 0.1 M HNO_3_ and 0.6 M KNO_3_ solution and recorded the electrochemical responses with differential pulse voltammetry (DPV) method with scanning range from −0.4 to 1.0 V with a 0.1 V/s scanning rate. Each sample was detected three times, and the results were calculated as mean ± RSD.

### 3.6. Detection of GPC3 Level in Human Serum Samples

To verify that the developed electrochemical nanobiosensor would be applied in serum detection, GPC3 in human serum samples was detected by standard addition methods under optimal conditions. Firstly, the human serum samples were obtained after approval from the Ethics Committee of Guangxi Key Laboratory of Metabolic Disease Research, 924th Hospital of the People’s Liberation Army Joint Logistics Support Force (Guilin, China). Three kinds of serum samples were prepared for determination by mixing 1.5 μL of normal serum with 1.5 μL of GPC3 solution (50.0 μg/mL, 80.0 μg/mL, and 100.0 μg/mL). The serum samples were detected as the above process instead of the GPC3 standard solution. The DPV of the electrochemical workstation was used for the determination. The measured concentration of GPC3 in human serum samples was calculated by the calibration line. Each sample was detected three times, and the results were calculated as mean ± RSD. 

## 4. Conclusions

In this study, one novel electrochemical nanobiosensor was constructed for the quantitative analysis of GPC3 based on H-rGO-Pd NPs nanozyme for signal amplification, combining GPC3_Apt_ and GPC3_Ab_ as recognition elements. In the presence of GPC3, both GPC3_Ab_ and H-rGO-Pd NPs-GPC3_Apt_ specifically bonded with GPC3 to form the H-rGO-Pd NPs-GPC3_Apt_/GPC3/GPC3_Ab_ sandwich-structure complex with a stable spatial structure as well as catalytic performance, which could enhance H_2_O_2_ to reduce the Ag ions in solution to metallic Ag, resulting in the deposition of Ag NPs on the surface of the biosensor. The amount of deposited Ag, which was derived from the amount of GPC3, was quantified by the DPV method. The developed nanobiosensor was able to determine GPC3 with the LOD of 3.30 ng/mL and showed good specificity, short-term stability, and recovery rates. Although the LOD value of the designed sensor was slightly higher, the GPC3 nanobiosensor can be an ideal solution for designing high-sensitivity clinical tests. We believe that this method may be an effective strategy for the determination of GPC3 with potential clinical applications and can be used to build accurate and simple sensors for other biomarkers. 

## Figures and Tables

**Table 1:** Detection of GPC3 by comparing analytical parameters with other methods.

Materials	Method	Linear Range	LOD	Sensitivity	References
AF2119/GPN_2_-NLuc	BLEIA	1.25–20 ng/mL	1.5 ng/mL	-	[4]
Immunoassay kit	ELISA	0.625–40 ng/mL	1.5 ng/mL	-	[10]
Anti-GPC3 McAb	TRFIA	1.0–50.0 ng/mL	0.039 ng/mL	-	[36]
RGO-Hemin/Au NPs/SPE	DPV	1.0–10.0 μg/mL	2.86 ng/mL	0.134 μA μM^−1^ cm^−2^	[35]
Ag/HGNs-Apt/GPC3/Apt/Au NPs/SPE	DPV	10–100 μg/mL	3.16 μg/mL	0.807 μA μM^−1^ cm^−2^	[37]
Ag/H-rGO-PdNPs-GPC3_Apt_/GPC3/GPC3_Ab_/AuNPs@rGO/SPE	DPV	0.01–100 μg/mL	3.30 ng/mL	1.535 μA μM^−1^ cm^−2^	This Work

BLEIA: bioluminescence enzyme immunoassay ELISA: enzyme-linked immunosorbent assay TRFIA: time-resolved fluorescence immunoassay DPV: differential pulse voltammetry.

**Table 2:** Analyses of the GPC3 electrochemical nanobiosensor proposed for the detection of GPC3 in human serum samples (Measured (n = 3)).

	Concentration of GPC3 Added (μg/mL)	Average GPC3 Concentration Measured (μg/mL)	Recovery (%)	RSD (%)
Normal human serum sample	25.0	26.63	106.52	8.81
	40.0	41.51	103.78	1.89
	50.0	51.95	103.90	6.96

## Data Availability

The data are available upon reasonable request.

## Supplementary Materials

The following supporting information can be downloaded at: https://www.mdpi.com/article/10.3390/molecules28052271/s1. Figure S1. (A) SEM image of H-rGO; (B) TEM image of H-rGO-Pd NPs. Figure S2. UV-vis spectrophotometer of GPC3_Apt_ (curve a), H-rGO-Pd NPs-GPC3_Apt_ (curve b), Supernate of H-rGO-Pd NPs-GPC3_Apt_ (curve c). Figure S3. (A) Effect of GPC3_Apt_ concentration on response current; (B) Effect of incubation temperature on response current; (C) Effect of incubation time on response current; (D) Effect of the amount of H-rGO-Pd NPs on response current (the concentration of GPC3 is 1.0 μg/mL). All above values are presented as the median from analysis of three independent experiments and the error bars indicate relative standard deviation. Figure S4. Reproducibility of GPC3 electrochemical nanobiosensor.

## References

[1] Yang J.D., Hainaut P., Gores G.J., Amadou A., Plymoth A., Roberts L.R. (2019). A global view of hepatocellular carcinoma: Trends, risk, prevention and management. Nat. Rev. Gastroenterol. Hepatol..

[2] Ren Z., Li A., Jiang J., Zhou L., Yu Z., Lu H., Zheng S. (2019). Gut microbiome analysis as a tool towards targeted non-invasive biomarkers for early hepatocellular carcinoma. Gut.

[3] Deng H., Shang W., Wang K., Guo K., Liu Y., Tian J., Fang C. (2022). Targeted-detection and sequential-treatment of small hepatocellular carcinoma in the complex liver environment by GPC-3-targeted nanoparticles. Nanobiotechnol. J..

[4] Yu S., Li Z., Li J., Zhao S., Wu S., Liu H., Hammock B.D. (2021). Generation of dual functional nanobody-nanoluciferase fusion and its potential in bioluminescence enzyme immunoassay for trace glypican-3 in serum. Sens. Actuators B Chem..

[5] Xia L., Teng Q., Chen Q., Zhang F. (2020). Preparation and Characterization of Anti-GPC3 Nanobody Against Hepatocellular Carcinoma. Int. Nanomed. J..

[6] Aydin Y., Koksal A.R., Thevenot P., Chava S., Heidari Z., Lin D., Dash S. (2021). Experimental Validation of Novel Glypican 3 Exosomes for the Detection of Hepatocellular Carcinoma in Liver Cirrhosis. J. Hepatocell. Carcinoma.

[7] Shin W.-R., Park D.-Y., Kim J.H., Lee J.-P., Thai N.Q., Oh I.-H., Kim Y.-H. (2022). Structure based innovative approach to analyze aptaprobe-GPC3 complexes in hepatocellular carcinoma. Nanobiotechnol. J..

[8] Du K., Li Y., Liu J., Chen W., Wei Z., Luo Y., Sui J. (2021). A bispecific antibody targeting GPC3 and CD47 induced enhanced antitumor efficacy against dual antigen-expressing HCC. Mol. Ther..

[9] Yu J.P., Xu X.G., Ma R.J., Qin S.N., Wang C.R., Wang X.B., Xu W.W. (2015). Development of a clinical chemiluminescent immunoassay for serum GPC3 and simultaneous measurements alone with AFP and CK19 in diagnosis of hepatocellular carcinoma. J. Clin. Lab. Anal..

[10] Tahon A.M., El-Ghanam M.Z., Zaky S., Emran T.M., Bersy A.M., El-Raey F., Johar D. (2019). Significance of Glypican-3 in Early Detection of Hepatocellular Carcinoma in Cirrhotic Patients. J. Gastrointest. Cancer.

[11] Yan J., Xiong H., Cai S., Wen N., He Q., Liu Y., Liu Z. (2019). Advances in aptamer screening technologies. Talanta.

[12] Dou B., Xu L., Jiang B., Yuan R., Xiang Y. (2019). Aptamer-functionalized and gold nanoparticle array-decorated magnetic graphene nanosheets enable multiplexed and sensitive electrochemical detection of rare circulating tumor cells in whole blood. Anal. Chem..

[13] Roguska A., Lesniewski A., Opallo M., Nogala W. (2021). Mediatorless electrocatalytic oxygen reduction with catalase on mercury-gold amalgam microelectrodes. Electrochem. Commun..

[14] Zhang X.Y., Liu S.G., Zhang W.J., Wang X.H., Han L., Ling Y., Luo H.Q. (2019). Photoelectrochemical platform for glucose sensing based on g-C3N4/ZnIn2S4 composites coupled with bi-enzyme cascade catalytic in-situ precipitation. Sens. Actuators B Chem..

[15] Chung S., Sicklick J.K., Ray P., Hall D.A. (2021). Development of a Soluble KIT Electrochemical Aptasensor for Cancer Theranostics. ACS Sens..

[16] Wei B., Zhong H., Wang L., Liu Y., Xu Y., Zhang J., Wang H. (2019). Facile preparation of a collagen-graphene oxide composite: A sensitive and robust electrochemical aptasensor for determining dopamine in biological samples. J. Biol. Macromol..

[17] Zhou X., Bai D., Yu H., Fu Y., Song L., Wu Y., Chen H. (2023). Detection of rare CTCs by electrochemical biosensor built on quaternary PdPtCuRu nanospheres with mesoporous architectures. Talanta.

[18] Gao T., Zhi J., Mu C., Gu S., Xiao J., Yang J., Xiang Y. (2018). One-step detection for two serological biomarker species to improve the diagnostic accuracy of hepatocellular carcinoma. Talanta.

[19] Sato F., Funo S., Cai Z., Chang G., He Y., Oyama M. (2021). Modification with platinum of silver-deposited nickel wire electrodes for electrocatalytic oxidation of alcohols. Electrochem. Commun..

[20] Bhatia A., Nandhakumar P., Kim G., Kim J., Lee N.-S., Yoon Y.H., Yang H. (2019). Ultrasensitive Detection of Parathyroid Hormone through Fast Silver Deposition Induced by Enzymatic Nitroso Reduction and Redox Cycling. ACS Sens..

[21] Mu Z., Tian J., Wang J., Zhou J., Bai L. (2022). A new electrochemical aptasensor for ultrasensitive detection of endotoxin using Fe-MOF and AgNPs decorated P-N-CNTs as signal enhanced indicator. Appl. Surf. Sci..

[22] Abedanzadeh S., Moosavi-Movahedi Z., Sheibani N., Moosavi-Movahedi A.A. (2022). Nanozymes: Supramolecular perspective. Biochem. Eng. J..

[23] Yaqoob S.B., Adnan R., Rameez Khan R.M., Rashid M. (2020). Gold, Silver, and Palladium Nanoparticles: A Chemical Tool for Biomedical Applications. Front. Chem..

[24] Vargas C., Simarro R., Reina J.A., Bautista L.F., Molina M.C., González-Benítez N. (2019). New approach for biological synthesis of reduced graphene oxide. Biochem. Eng. J..

[25] Huang N., Xu E., Xie J., Liu Y., Deng Z., Wang J., Ye Q. (2022). A sliver deposition signal-enhanced optical biomolecular detection device based on reduced graphene oxide. Talanta.

[26] Guo L., Bai J., Li C., Meng Q., Liang H., Sun W., Liu H. (2013). A novel catalyst containing palladium nanoparticles supported on PVP composite nanofiber films: Synthesis, characterization and efficient catalysis. Appl. Surf. Sci..

[27] Zhang J., Xu Q., Pei W., Cai L., Yu X., Jiang H., Chen J. (2021). Self-assembled recombinant camel serum albumin nanoparticles-encapsulated hemin with peroxidase-like activity for colorimetric detection of hydrogen peroxide and glucose. Int. J. Biol. Macromol..

[28] Dong J., Yang H., Zhao J., Wen L., He C., Hu Z., Hou C. (2022). Sandwich-type microRNA biosensor based on graphene oxide incorporated 3D-flower-like MoS2 and AuNPs coupling with HRP enzyme signal amplification. Mikrochim. Acta.

[29] Tsounis C., Subhash B., Kumar P.V., Bedford N.M., Zhao Y., Shenoy J., Amal R. (2022). Pt Single Atom Electrocatalysts at Graphene Edges for Efficient Alkaline Hydrogen Evolution. Adv. Funct. Mater..

[30] Zhou H., Chen L., Li S., Huang S., Sun Y., Chen Y., Li X. (2020). One-step electroreduction preparation of multilayered reduced graphene oxide/gold-palladium nanohybrid as a proficient electrocatalyst for development of sensitive hydrazine sensor. J. Colloid Interface Sci..

[31] Li J., Si Y., Park Y.E., Choi J.-S., Jung S.M., Lee J.E., Lee H.J. (2021). A serotonin voltammetric biosensor composed of carbon nanocomposites and DNA aptamer. Microchim. Acta.

[32] Amirjani A., Kamani P., Hosseini H.R.M., Sadrnezhaad S. (2022). SPR-based assay kit for rapid determination of Pb^2+^. Anal. Chim. Acta.

[33] Baghayeri M., Nodehi M., Amiri A., Amirzadeh N., Behazin R., Iqbal M.Z. (2020). Electrode designed with a nanocomposite film of CuO Honeycombs/Ag nanoparticles electrogenerated on a magnetic platform as an amperometric glucose sensor. Anal. Chim. Acta.

[34] Yan H., He B., Zhao R., Ren W., Suo Z., Xu Y., Liu R. (2022). Electrochemical aptasensor based on Ce_3_NbO_7_/CeO_2_@ Au hollow nanospheres by using Nb. BbvCI-triggered and bipedal DNA walker amplification strategy for zearalenone detection. J. Hazard. Mater..

[35] Li G., Feng H., Shi X., Chen M., Liang J., Zhou Z. (2021). Highly sensitive electrochemical aptasensor for Glypican-3 based on reduced graphene oxide-hemin nanocomposites modified on screen-printed electrode surface. Bioelectrochemistry.

[36] Chen J.-J., Xie C.-M., Wang C.-R., Wan Y., Dong Z.-N., Li M., Xu W.-W. (2017). Development of a Time-Resolved Fluorescence Immunoassay for the Diagnosis of Hepatocellular Carcinoma Based on the Detection of Glypican-3. J. Fluoresc..

[37] Zhou Z., Zhao L., Li W., Chen M., Feng H., Shi X., Li G. (2020). Glypican-3 electrochemical aptamer nanobiosensor based on hemin/graphene nanohybrids peroxidase-like catalytic silver deposition. Mikrochim. Acta.

[38] Li G., Li H., Chen W., Chen H., Wu G., Tan M., Zhou Z. (2021). Highly Sensitive Electrochemical Aptasensor for Detection of Glypican-3 Using Hemin-Reduced Graphene Oxide-Platinum Nanoparticles Coupled with Conductive Reduced Graphene Oxide-Gold Nanoparticles. J. Biomed. Nanotechnol..

[39] Jin H., Zhao C., Gui R., Gao X., Wang Z. (2018). Reduced graphene oxide/nile blue/gold nanoparticles complex-modified glassy carbon electrode used as a sensitive and label-free aptasensor for ratiometric electrochemical sensing of dopamine. Anal. Chim. Acta.

[40] Peng G., Yu Y., Chen X., Huang H. (2020). Highly sensitive amperometric α-ketoglutarate biosensor based on reduced graphene oxide-gold nanocomposites. Int. J. Anal. Chem..

[41] Patella B., Sortino A., Mazzara F., Aiello G., Drago G., Torino C., Inguanta R. (2021). Electrochemical detection of dopamine with negligible interference from ascorbic and uric acid by means of reduced graphene oxide and metals-NPs based electrodes. Anal. Chim. Acta.

[42] Wu M., Liu S., Qi F., Qiu R., Feng J., Ren X., Pan H. (2022). A label-free electrochemical immunosensor for CA125 detection based on CMK-3 (Au/Fc@MgAl-LDH) n multilayer nanocomposites modification. Talanta.

