# Supplementary material for: H-rGO-Pd NPs Nanozyme Enhanced Silver Deposition Strategy for Electrochemical Detection of Glypican-3
Source: Molecules. 2023 Feb 28;28(5):2271. doi: 10.3390/molecules28052271 (PMC10004744; doi:10.3390/molecules28052271)
Supplement: Supplementary file 1 [file molecules-28-02271-s001.zip › molecules-2176011-supplementary.pdf]

Electronic Supplementary Materisls

H-rGO-Pd NPs Nanozyme Enhanced Silver Deposition Strategy for Electrochemical Detection of Glypican-3

Guiyin Li [1,2], Bo Wang [1], Ling Li [1], Xinhao Li [1], Ruijie Yan [1], Jintao Liang [1], Xinchun Zhou [3,*], Liuxun Li [4,*] and Zhide Zhou [1,*]

1 Guangxi Key Laboratory of Information Materials, School of Life and Environmental Sciences, Guilin University of Electronic Technology, Guilin 541004, China
2 College of Chemistry, Guangdong University of Petrochemical Technology, Guandu Road, Maoming 525000, China
3 Guangdi Maoming Chemical Co., Ltd., Maoming High-Tech Industrial Development Zone, Maoming 525000, China
4 Solid Tumour Target Discovery Laboratory, Translational and Clinical Research Institute, Newcastle University Centre for Cancer, Faculty of Medical Sciences, Newcastle University, Newcastle upon Tyne NE2 4HH, UK
* Correspondence: xczhou2009@163.com (X.Z.); l.li32@newcastle.ac.uk (L.L.); zzdlgy@163.com (Z.Z.)

1. Characterization of H-rGO-Pd NPs

As shown in Figure S1A, the folding membrane structure of G on H-rGO shows that H-rGO is synthesized successfully. As shown in Figure S1B, the surface of H-rGO-Pd NPs exhibited uniformly shaped dark particles, i.e., Pd NPs attached to its surface, which indicated that H-rGO-Pd NPs had been successfully prepared.

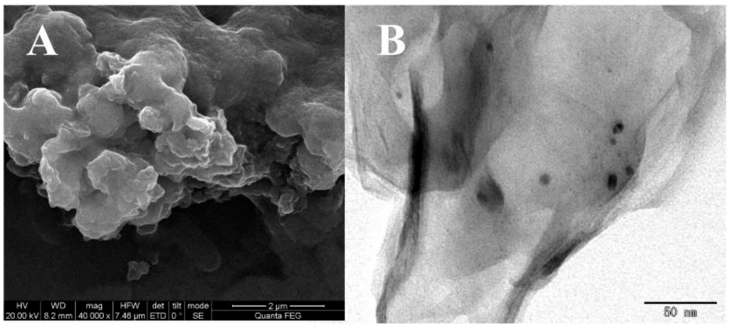

Figure S1. (A)SEM image of H-rGO; (B) TEM image of H-rGO-Pd NPs.

2. Characterization of H-rGO-Pd NPs-GPC3$_{Apt}$ detection probe

To explore whether the combination of GPC3$_{Apt}$ and H-rGO-Pd NPs was successful, the H-rGO-Pd NPs-GPC3$_{Apt}$ was characterized and analyzed by UV-vis

(Figure S2). 10 μmol/L $GPC3_{Apt}$ solution, 0.05 mg/mL H-rGO-Pd NPs-$GPC3_{Apt}$ solution, and centrifuged H-rGO-Pd NPs-$GPC3_{Apt}$ supernatant were each 200 μL, and the absorbance of the materials were characterized and analyzed by UV-vis s at wavelength (200 nm-500 nm). $GPC3_{Apt}$ has a prominent absorption peak at wavelength 260 nm, while H-rGO-Pd NPs-$GPC3_{Apt}$ has prominent absorption peaks at wavelength 262 nm. It was significantly stronger than the absorption peaks of H-rGO-Pd NPs-$GPC3_{Apt}$ supernatant at 265 nm. According to this result, $GPC3_{Apt}$ was successfully cbound to H-rGO-Pd NPs with 80% binding rate.

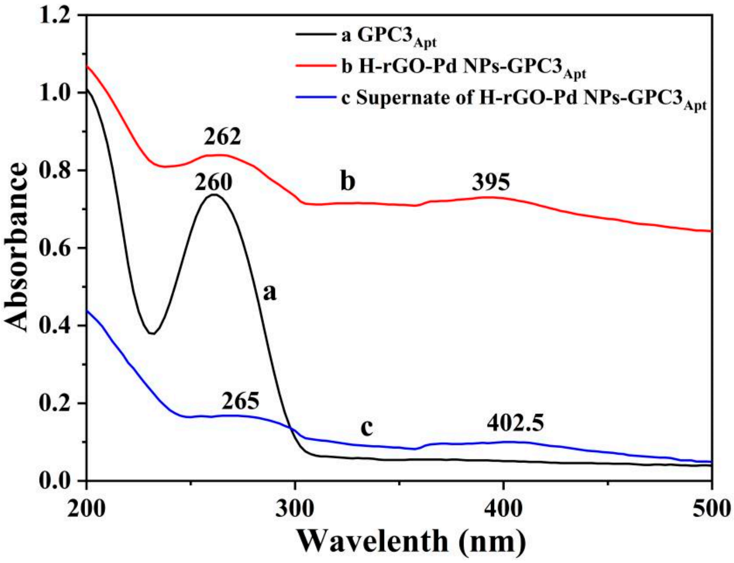

Figure S2. UV-vis spectrophotometer of $GPC3_{Apt}$ (curve a), H-rGO-Pd NPs-$GPC3_{Apt}$ (curve b), Supernate of H-rGO-Pd NPs-$GPC3_{Apt}$ (curve c)

3. Optimization of conditions for GPC3 electrochemical nanobiosensor

In order to optimize the performance of the sensor, the experimental conditions of $GPC3_{Apt}$ concentration, GPC3 incubation time, incubation temperature, and H-rGO-Pd NPs-$GPC3_{Apt}$ dosage were optimized by the single-factor variable method, and DPV measurements were performed on the sensors under different conditions.

The GPC3 aptamer is intrinsically bound to GPC3, so changes in the aptamer concentration will influence the corresponding electrochemical nanobiosensor of GPC3 (Figure S3A). With all other factors constant, when the $GPC3_{Apt}$ concentration ranged from 0.1 to 5.0 μmol/L, the nanobiosensor response current increased, and the maximum value occurd when the concentration of $GPC3_{Apt}$ was 5.0 μmol/L. There was a drop in sensor response current when $GPC3_{Apt}$ concentration was from 5.0 μmol/L to 6.0 μmol/L. Accordingly, 5.0 μmol/L of $GPC3_{Apt}$ was optimal.

Figure S3B showed how incubation temperature affects electrochemical nanobiosensor performance for GPC3 (4°C-37°C). During incubation at 4°C-25°C, the sensor response current continued to increase, and the maximum value occurs at the incubation temperature of 25°C. Incubation temperatures of 25°C-37°C, the response current of the sensor decreased. Therefore, the optimal incubation temperature was 25°C.

As seen in Fig.S3C, the electrochemical nanobiosensor GPC3 responded differently with the incubation time (from 10 min to 120 min). Incubating GPC3 for 10 min to 60 min significantly increased its sensor response current and peaks at 30 minutes. When the incubation time was 60 min to 120 min, the nanobiosensor response current continued to decrease. Thus, 60 min of incubation was chosen.

As shown in Fig.S3D, the electrochemical nanobiosensor was affected by the amount of H-rGO-Pd NPs (1.0 μL-7.0 μL). When the amount of H-rGO-Pd NPs increased from 1.0 μL to 4.0 μL, a more muscular response current was generated by the sensor. And when the amount was from 4.0 μL to 7.0 μL, the sensor response

current slowly decreased and gradually flattend out. Therefore, 4.0 μL was the ideal dosage for H-rGO-Pd NPs nanozyme.

According to the results of the experiments, the following conditions were optimal: (a) $GPC3_{Apt}$ concentration: 5.0 μmol/L; (b) temperature for incubation: 25°C; (c) incubation time: min; (d) the amount of H-rGO-Pd NPs nanozyme: 4.0 μL.

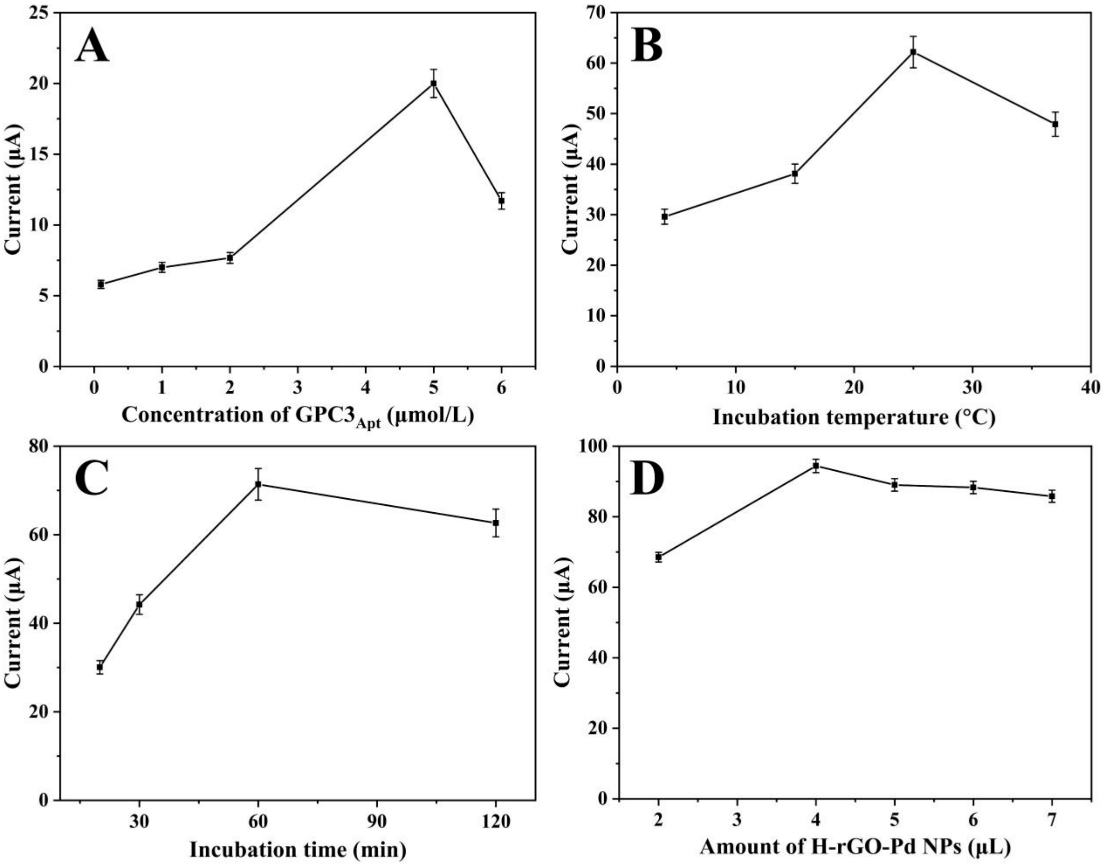

Figure S3. (A) Effect of $GPC3_{Apt}$ concentration on response current. (B) Effect of incubation temperature on response current. (C) Effect of incubation time on response current. (D) Effect of the amount of H-rGO-Pd NPs on response current (the concentration of GPC3 is 1.0 μg/mL). All above values are presented as the median from analysis of three independent experiments and the error bars indicate relative standard deviation.

4. Reproducibility of GPC3 electrochemical aptasensor

To further examine that the results are reproducible, five parallel of nanobiosensors added with 1.0 μg/mL GPC3 was assessed. The relative standard deviations of the response currents of five sensors (25.3 μA, 26.7 μA, 25.5 μA, 24.8 μA, and 25.1 μA) was 2.56%, suggesting that the GPC3 electrochemical nanobiosensor can be reproducible.

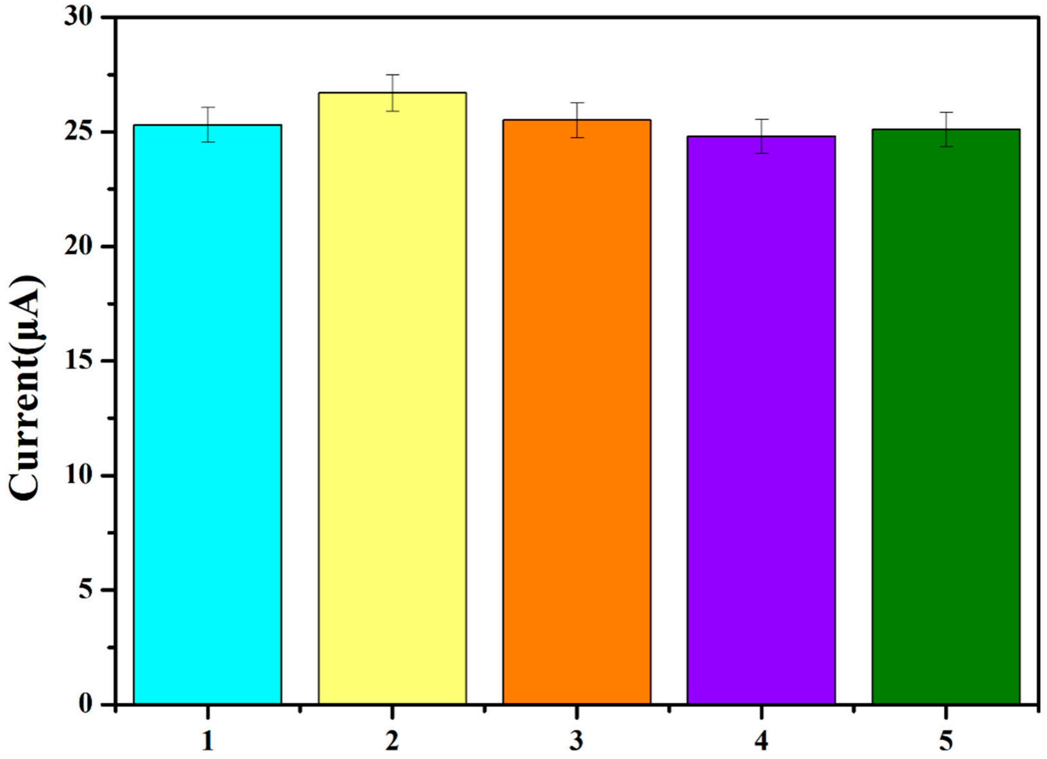

Figure S4. Reproducibility of GPC3 electrochemical nanobiosensor
